# Case report: Corneal endothelial degeneration and optic atrophy in dentatorubral-pallidoluysian atrophy quantified by specular micrography and optical coherence tomography

**DOI:** 10.3389/fneur.2022.953787

**Published:** 2022-09-13

**Authors:** Shunya Takizawa, Hiroto Mitamura, Yuko Ohnuki, Kenji Kawai, Yoichi Ohnuki, Eiichiro Nagata, Wakoh Takahashi

**Affiliations:** ^1^Department of Neurology, Tokai University Oiso Hospital, Oiso, Japan; ^2^Department of Ophthalmology, Keio University, Tokyo, Japan; ^3^Department of Ophthalmology, Kawasaki Municipal Hospital, Kawasaki, Japan; ^4^Department of Medical Ethics, Tokai University School of Medicine, Isehara, Japan; ^5^Department of Ophthalmology, Tokai University Oiso Hospital, Oiso, Japan; ^6^Department of Neurology, Tokai University School of Medicine, Isehara, Japan

**Keywords:** CAG repeat, corneal endothelial degeneration, dentatorubral-pallidoluysian atrophy, optical coherence tomography, specular micrography

## Abstract

**Introduction:**

Dentatorubral-pallidoluysian atrophy (DRPLA) is an autosomal dominant neurodegenerative disease with various neurological manifestations. Corneal endothelial degeneration and optic atrophy have been reported separately; however, there are no reports of corneal endothelial degeneration with optic atrophy.

**Cases:**

Herein, we present four related patients with DRPLA: two patients (69-year-old woman and 80-year-old man) who exhibited both corneal endothelial degeneration and optic atrophy and another two (49- and 51-year-old women, respectively) who exhibited only corneal endothelial degeneration. We quantified the reduction in corneal endothelial cell density (ECD) and hexagonality using specular microscopy and thinning of the circumpapillary retinal nerve fiber layer (RNFL) using optical coherence tomography (OCT).

**Conclusion:**

This is the first report of DRPLA accompanied by corneal endothelial degeneration and/or optic atrophy, which were both quantified based on the corneal ECD and the circumpapillary RNFL thickness using specular micrography and OCT, respectively. The pathophysiological mechanism is unclear; however, the involvement of the nuclear receptor TLX interacting with atrophin-1 may be implicated in ophthalmic manifestations of DRPLA. Therefore, we recommend performing specular micrography and/or OCT when patients with DRPLA experience visual disturbances.

## Introduction

Dentatorubral-pallidoluysian atrophy (DRPLA) is an autosomal dominant neurodegenerative disease characterized by cerebellar ataxia, choreoathetosis, myoclonus, epilepsy, dementia, and psychiatric symptoms (1). Only a few reports have shown ophthalmic manifestations in patients with DRPLA. Corneal endothelial degeneration (2–4) and optic atrophy (5) have been reported separately; however, there are no reports of corneal endothelial degeneration and optic atrophy simultaneously in these patients. Herein, we present four patients from a single family with DRPLA and corneal endothelial degeneration and/or optic atrophy.

Quantitative ophthalmological examinations are crucial to evaluate the corneal and optic nerve lesions in neurological diseases. Specular micrography can qualitatively describe the cellular morphology and quantify the endothelial cell damage (6). Essentially, specular microscopy automatically measures the corneal endothelial cell density (ECD), coefficient of variation (CV), and hexagonality (HEX). Optical coherence tomography (OCT) (7) can assess the ocular fundus, as well as the circumpapillary retinal nerve fiber layer (RNFL). This is the first study to quantify both corneal ECD and RNFL in patients with DRPLA.

## Case description

**Figure 1:**
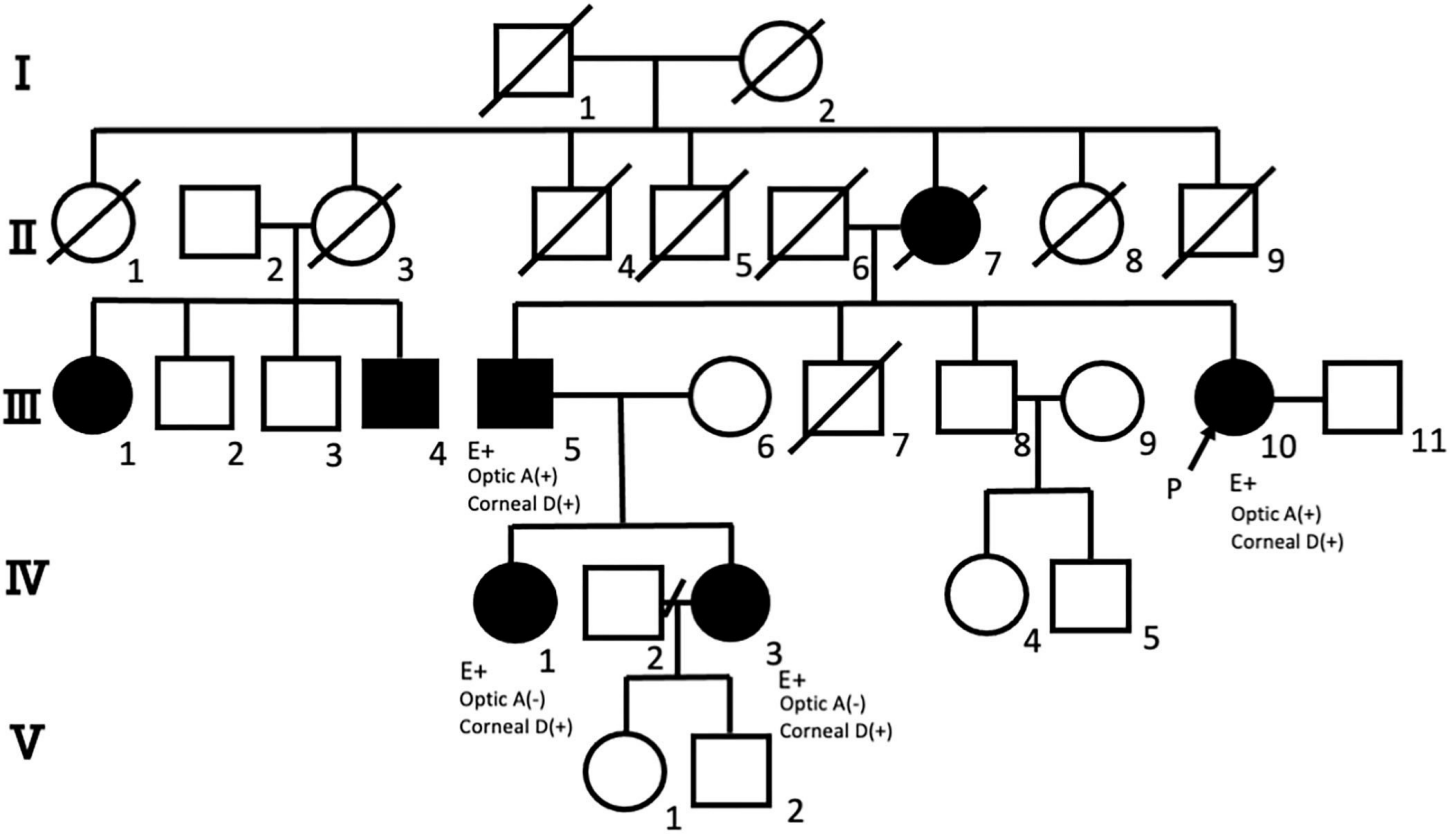
Pedigree of the family with dentatorubral-pallidoluysian atrophy (DRPLA), including the four patients in this study. Pedigree of the family with DRPLA showing generations (*Roman numerals*) of affected (*solid symbols*) and unaffected (*open symbols*) members. Roman numerals represent generation numbers. Squares indicate male members; circles, female members; P, proband; oblique, deceased; optic A, optic atrophy; corneal D, corneal endothelial degeneration. Genetic analysis (E+) shows the expanded allele of CAG repeats at the DRPLA locus (III-5, III-10, IV-1, and IV-3).

The pedigree of a single Japanese family with DRPLA, including the following four patients, is presented in Figure 1. The proband was a 69-year-old Japanese woman (III-10) whose family history revealed that her mother (II-7) experienced gait disturbances in her 80s and died at 93 years old. In addition, she noticed progressive worsening of vision, even after cataract surgery. Her cousin (III-1) died at 80 years old of an unknown cause and suffered from a psychotic disorder for many years. The proposita and affected relatives (III-5, IV-3, and IV-1) are presented in detail in the following subsections.

### Patient 1 (III-10)

**Figure 2:**
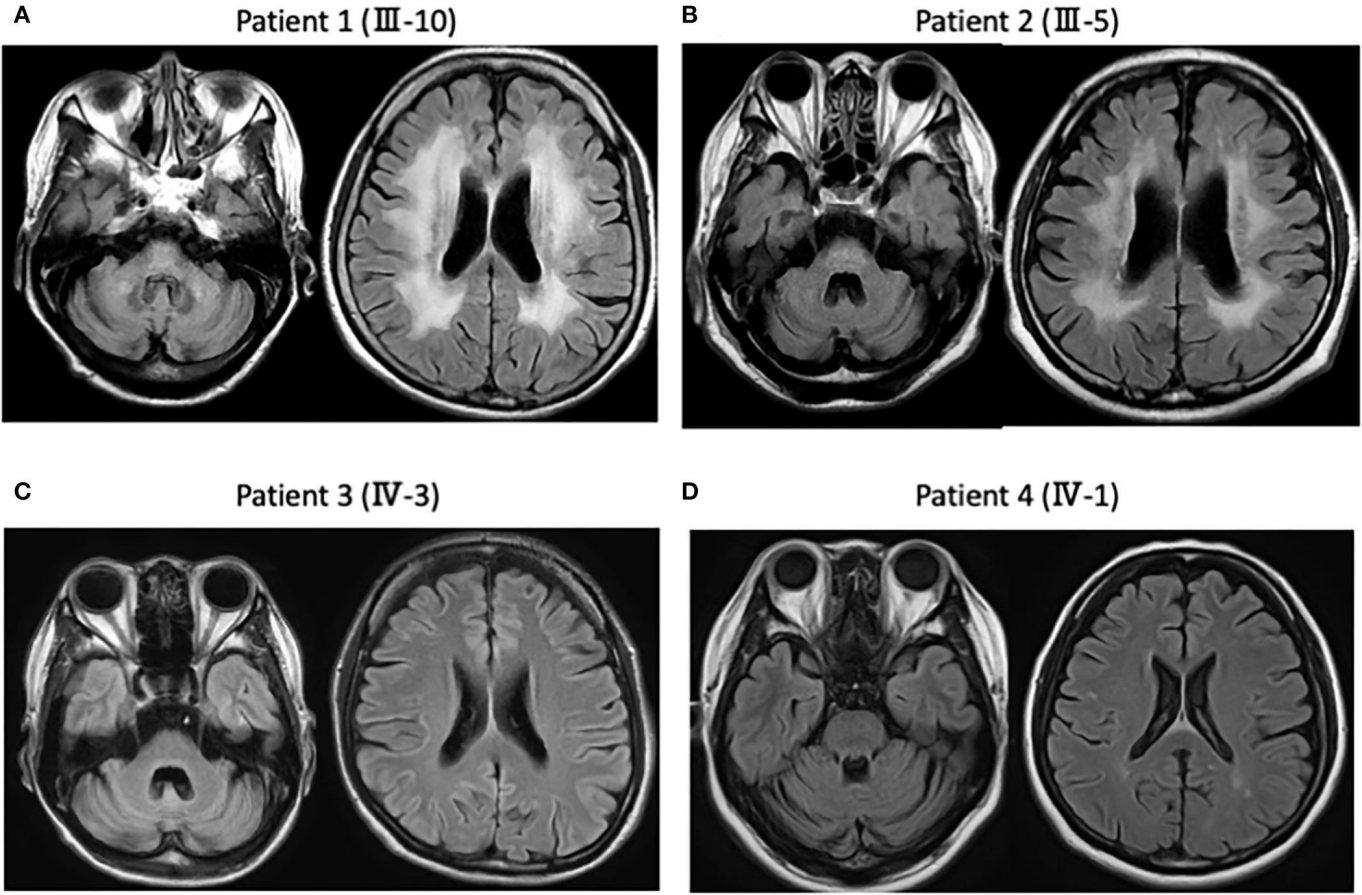
Magnetic resonance imaging (MRI) findings in the present four patients. **(A)** Patient 1 (III-10). Fluid attenuated inversion recovery images of the MRI show diffuse cerebellar and brainstem atrophy, as well as high-intensity signals in the diffuse periventricular white matter. **(B)** Patient 2 (III-5). MRI shows diffuse cerebellar and brainstem atrophy, as well as high-intensity signals in the diffuse periventricular white matter. **(C)** Patient 3 (IV-3). MRI shows diffuse cerebellar and brainstem atrophy without white matter changes. **(D)** Patient 4 (IV-1). MRI shows diffuse cerebellar atrophy without periventricular white matter changes.

A 69-year-old Japanese woman gradually noticed gait disturbance and dysarthria at 61 years old and, in addition to these symptoms, experienced visual disturbances and consequently required a cane while walking. Neurological examinations showed a bilateral reduction in visual acuity, dysarthria, ataxic gait, coordination disturbances in the upper and lower extremities, and pyramidal tract signs, with neither dementia nor myoclonus. Fluid-attenuated inversion recovery images of the MRI showed cerebellar and brainstem atrophy and high-intensity signals in diffuse periventricular white matter (Figure 2A). Genetic analysis revealed that the expanded allele had 55 CAG repeats at the *DRPLA* locus, whereas the normal allele contained 15 repeats.

**Figure 3:**
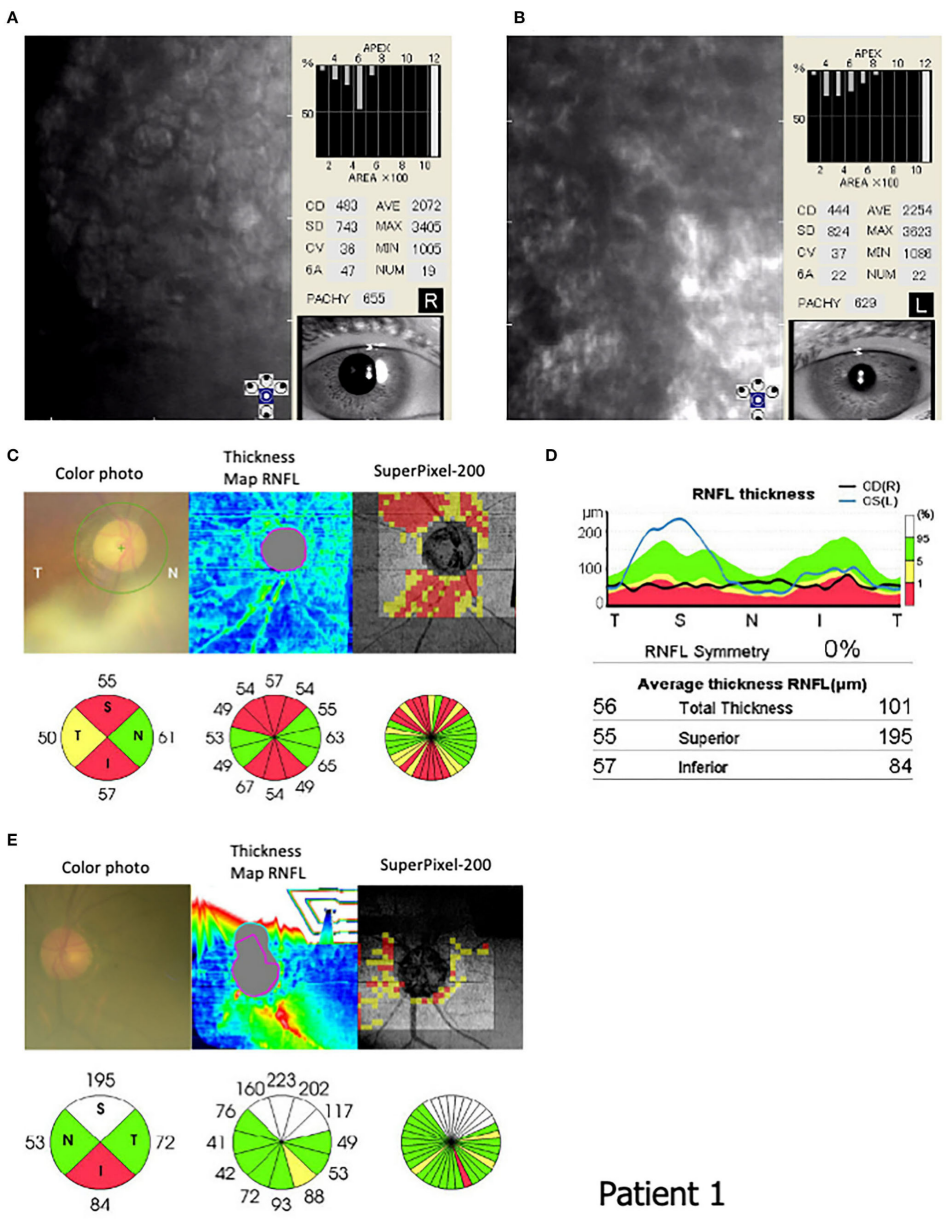
Endothelial corneal cell density (ECD) and circumpapillary retinal nerve fiber layer (RNFL) in Patient 1 (III-10). Specular microscopy (FA-3609, Konan Medical, Inc. Japan) shows **(B)** corneal guttata, **(A,B)** pleomorphic cellular patterns of endothelial structures, and **(B)** rounded dark cells with light borders. The corneal cell density is 483 and 444 cells/mm^2^ in the **(A)** right and **(B)** left eyes, respectively. **(C–E)** An optical coherence tomography (OCT: 3D CT-2000, Topcon Medical Japan, Co. Ltd, Japan) indicates the ocular fundus color photograph, RNFL thickness map, super Pixel-200 image, and RNFL values in the circular areas. An OCT shows RNFL thinning in the superior (55 μm) and inferior (57 μm) areas of the **(C)** right eye and inferior area (84 μm) of the **(E)** left eye.

The patient's past ocular history included cataract surgery with lens implantation in the right eye at 67 years old. At our examination, the best corrected visual acuity was 20/200 and 20/20, and the intraocular pressure was maintained at 10 and 13 mmHg in the right and left eyes, respectively. A slit-lamp examination showed corneal swelling and guttata without pigmentation in the left eye but not in the right. Specular microscopy showed increased corneal thickness, multiple corneal guttata, pleomorphic cellular patterns of endothelial structures, and rounded dark cells with light borders (Figures 3A,B). Quantitatively, corneal ECD had severely decreased to 483 and 444 cells/mm^2^, CV was 36 and 37%, and HEX had decreased to 47 and 22% in the right and left eyes, respectively. In addition, the OCT showed RNFL thinning in the superior (55 μm) and inferior (57 μm) areas of the right eye and inferior area (84 μm) of the left eye, corresponding to optic nerve atrophy (Figures 3C–E).

### Patient 2 (III-5)

An 80-year-old man noticed a gait disturbance at 60 years of age, with the gait disturbance gradually progressing over 10 years; consequently, he needed a wheelchair. Neurological examinations revealed dysarthria, ataxic gait, and disturbance of coordination in the upper and lower extremities with no dementia, myoclonus, or spasticity. An MRI showed cerebellar and brainstem atrophy and high-intensity signals in the periventricular white matter (Figure 2B). Genetic analysis revealed that the expanded allele had 55 CAG repeats at the *DRPLA* locus, whereas the normal allele contained 17 repeats.

The patient's past ocular history included cataract surgery with lens implantation in the right eye at 70 years old and in the left eye at 75 years old. At our examination, visual acuity was 20/100 in both eyes, and intraocular pressure was normal (10 mmHg) in both eyes. A slit-lamp examination showed no corneal guttata or swelling in both eyes. However, the specular microscopy revealed corneal guttata and pleomorphic cellular patterns of endothelial structures in both eyes (Supplementary Figures 1A,B). Corneal ECD had decreased to 562 and 1,675 cells/mm^2^, CV to 29 and 36%, and HEX to 38 and 34% in the right and left eyes, respectively. In addition, the OCT revealed RNFL thinning in the right eye (average RNFL thickness: 46 μm), corresponding to optic nerve atrophy (Supplementary Figure 1C).

### Patient 3 (IV-3)

A 49-year-old woman developed gait ataxia and intellectual disability in her 30s and experienced generalized tonic-clonic seizures at 35 years old. The expanded allele at the *DRPLA* locus was confirmed when the patient was 42 years old in our affiliated hospital. She visited our hospital at 49 years old and showed progressive dementia, dystonia, and ataxic gait with neither myoclonus nor spasticity. An MRI showed diffuse cerebellar and brainstem atrophy without white matter changes (Figure 2C).

The patient had no past ocular history, and visual acuity was 20/200 in both eyes; however, it was unreliable because of dementia. The intraocular pressure was normal (15 and 16 mmHg in the right and left eyes, respectively). A slit-lamp examination showed no corneal guttata or swelling in both eyes. On the other hand, the specular microscopy showed multiple corneal guttata and rounded dark cells with light borders in the left eye and pleomorphic cellular patterns of endothelial structures in both eyes (Supplementary Figure 2). Corneal ECD had decreased to 2,326 and 1,637 cells/mm^2^, CV was 38 and 33%, and HEX was 25 and 81% in the right and left eyes, respectively. Optic atrophy was not observed photographically. The OCT was not examined because she could not maintain visual fixation owing to dementia. Additionally, the visual evoked potential was normal.

### Patient 4 (IV-1)

A 51-year-old woman has no observed abnormalities, except mild ataxia in the lower extremities, during neurological examination. An MRI revealed diffuse cerebellar atrophy without periventricular white matter changes (Figure 2D). Genetic analysis revealed that the expanded allele had 58 CAG repeats at the *DRPLA* locus, whereas the normal allele contained 17 repeats.

The patient's past ocular history included laser *in situ* keratomileusis surgery when she noticed a visual disturbance at 49 years old and visited the Department of Ophthalmology at another Hospital. The patient's visual acuity was 20/50 and 20/200 in the right and left eyes, respectively. The intraocular pressure was normal (12 and 13 mmHg in the right and left eyes, respectively). A slit-lamp examination showed no corneal guttata but slight corneal edema in both eyes. The specular microscopy showed no corneal guttata or rounded dark cells. Corneal ECD had decreased to 917 and 532 cells/mm^2^, CV to 46 and 34%, and HEX to 23 and 39% in the right and left eyes, respectively (Supplementary Figures 3A,B). The OCT showed thinning of the RNFL in the inferior part of the optic nerve papilla (Supplementary Figures 3C,D); however, this was unreliable because the optic disk was tilted owing to high myopia. Therefore, she was diagnosed with bullous keratopathy due to corneal endothelial degeneration. We pathologically examined Descemet's membrane and the corneal endothelial cells after Descemet's stripping endothelial keratoplasty, but no specific pathological abnormality was observed. The patient eventually underwent corneal transplantation in the left eye, and the left visual acuity recovered to 20/20.

## Discussion

In this study, we described four patients with DRPLA: two (Patients 1 and 2 in Table 1) who exhibited both corneal endothelial degeneration and optic atrophy and two (Patients 3 and 4 in Table 1) who exhibited only corneal endothelial degeneration. Our patients had no history of trauma, inflammation, glaucoma, or contact lens use; however, Patients 1, 2, and 4 had a history of ophthalmologic surgery. Patient 4, who is phakic, was first diagnosed with bullous keratopathy due to corneal endothelial degeneration. She then received cataract surgery in her left eye prior to corneal transplantation. Therefore, the effect of ophthalmologic surgery on corneal endothelial cells was not considered. Patients 1, 2, and 3 showed corneal guttata, which may have influenced the reliability of the specular microscopy through swelling of the cornea. However, we could estimate the counts of ECD, CV, and HEX in the specular microscopy, which are valuable enough to evaluate the corneal morphology despite the swollen cornea. Furthermore, comorbidities such as Fuchs' endothelial corneal dystrophy are unlikely in our four patients because specific corneal pigmentation was not observed in the slit-lamp examination. Patient 4 underwent a pathologic examination of her endothelium and did not have the classic excrescences of Fuchs' dystrophy; therefore, this was a different process than Fuchs' dystrophy.

**Table 1:** Summary of patients with dentatorubral-pallidoluysian atrophy (DRPLA) accompanied by ophthalmic lesions, including the four present patients.

**Reference number**	**Our report**	**Our report**	**Our report**	**Our report**	**Ref 2**	**Ref 2**	**Ref 3**	**Ref 3**	**Ref 4**	**Ref 5**
	Patient 1	Patient 2	Patient 3	Patient 4	Case 1 (2)	Case 2 (2)	Case 1 (3)	Case 2 (3)	Case 1 (4)	Case 1 (5)
Age (years)	69	80	49	51	46	39	52	69	35	47
Sex	Female	Male	Female	Female	Female	Female	Male	Male	Male	Male
Age of onset of symptoms	Gait disturbance and dysarthria at 61 y/o	Ataxia at 60 y/o	Ataxia and epilepsy at 35 y/o	Visual disturbance at 49 y/o	Gait disturbance and ataxia at 36 y/o	Ataxia at 36 y/o	Gait disturbance and dysarthria at 36 y/o	Gait disturbance at 61 y/o	Ataxia at around 25 y/o	Progressive visual loss at 47 y/o
Family History	7 affected patients in the pedigree of the family	7 affected patients in the pedigree of the family	7 affected patients in the pedigree of the family	7 affected patients in the pedigree of the family	9 patients	9 patients	2 patients	3 patients	4 patients	1 patient (no family Hx)
Number of CAG repeats	55/15	55/17	Increased	58/17	67/20	66/19	62/18	56/11	71/21	62/12
Cerebellar ataxia	Positive	Positive	Positive	Slightly positive	Positive	Positive	Positive	Positive	Positive	Positive
Choreoathetosis / dystonia	Negative	Negative	Positive	Negative	Not described	Not described	Negative	Not described	Not described	Positive
Pyramidal tract sign	Positive	Negative	Negative	Negative	Not described	Not described	Positive	Not described	Positive	Negative
Myoclonus	Negative	Negative	Negative	Negative	Not described	Not described	Negative	Not described	Positive	Negative
Dementia	Negative	Negative	Positive with epilepsy	Negative	Not described	Not described	Positive	Not described	Positive	Negative
MRI findings in the cerebrum	White matter changes (+)	White matter changes (+)	White matter changes (–)	White matter changes (–)	Not described	Not described	Not described	Not described	Not described	White matter changes (+)
MRI findings in the infratentorial region	Brainstem and cerebellar atrophy	Brainstem and cerebellar atrophy	Brainstem and cerebellar atrophy	Cerebellar atrophy	Not described	Not described	Cerebellar atrophy	Cerebellar atrophy	Cerebrum and cerebellar atrophy	Cerebellar atrophy
Corneal endothelial cell density										
Right eye	483	562	2,326	917	762	951	500	Not described	876	Not described*
Left eye	444	1,675	1,637	532	540	866	500	1,506	941	Not described*
Optic atrophy	Positive in both eyes by OCT	Positive in the right eye by OCT	Negative	Negative	Negative	Negative	Negative	Negative	Negative	Positive in both eyes
Remarks	A cane needed	A wheelchair needed	A wheelchair needed	Corneal transplantation						*Only corneas with 2–3+ guttata were described

Hx, history; MRI, magnetic resonance imaging; OCT, optical coherence tomography.

*Not described* means that corneal endothelial cell density in right and left eyes in specular microscopy was not examined, but corneas with 2−3+ guttata in conventional slit-lamp examination were described.

We summarized the clinical characteristics, genetic analysis, MRI findings, and ophthalmological examination in the four patients and those with ophthalmologic lesions previously reported (2–5) in Table 1. These patients showed typical signs similar to those of DRPLA patients without ophthalmological lesions. There was no relationship between corneal endothelial degeneration and/or optic atrophy and the number of CAG repeats at the *DRPLA* locus in our four patients. Although Patient 4 showed mild ataxia in the lower extremities and obvious corneal endothelial degeneration, the number of CAG repeats was almost the same as that in our other patients. Patients 1 and 2, who had both corneal endothelial degeneration and optic atrophy, as well as white matter changes on MRI, were aged persons. As previously reported (8), white matter changes on MRI are unsurprisingly related to aging and the size of the expanded CAG repeat in patients with DRPLA. Since the patient with optic atrophy (5) was not aged, this ophthalmic manifestation may be influenced by other specific factors, such as the *DRPLA* gene product atrophin-1 (9, 10).

We recommend that all patients with DRPLA undergo a detailed ophthalmological examination, including OCT and specular micrography, when possible. A specular micrograph can present qualitative cellular morphology and quantitative endothelial cell damage by determining the corneal ECD. Corneal endothelial cell loss is compensated by increased cell size and decreased cell density (6). A Japanese population-based cross-sectional study, enrolling 5,713 participants (62.8 ± 10.3 years old), determined the mean ECD (men: 2,762 ± 241, women: 2,719 ± 234 cells/mm^2^), CV (men: 41.55 ± 6.70%, women: 43.29 ± 6.63%), and HEX (men: 47.25 ± 7.20%, women: 44.77 ± 7.14%) of the cohort (11). Compared with these normal values, corneal ECD and HEX in our patients were reduced in addition to corneal guttata and rounded dark cells with light borders corresponding to corneal endothelial cell damage. Silver et al. (5) simply described the presence of corneas with 2–3+ guttata in slit-lamp examinations without showing any pictures; however, a slit-lamp examination cannot accurately detect corneal endothelial damage like specular micrography at high magnification can (6).

Optical coherence tomography can analyze specific retina structures and provide high-resolution cross-sectional and volumetric images in the ocular fundi. In particular, RNFL measurements can provide objective evidence of nerve swelling or atrophy. Although one report has presented a patient with DRPLA accompanied by optic atrophy (6), optic atrophy was confirmed only by fundoscopic examination rather than RNFL measurement. Conversely, OCT was performed only in spinocerebellar ataxia type 1, showing prominent RNFL reduction in temporal areas with high statistical significance (12). However, we did not observe selective RNFL reduction in temporal areas in our patients with DRPLA.

The pathophysiological mechanism underlying corneal endothelial cell loss and optic nerve atrophy in patients with DRPLA is unclear. We found no specific pathological abnormality in Descemet's membrane or the corneal endothelial cells in Patient 4. Ito et al. (3) found that corneal endothelial cells, derived from the neuroectoderm, clearly express DRPLA protein, suggesting that mutant DRPLA protein may be directly related to corneal endothelial and neuronal degeneration. Recently, the nuclear receptor TLX, an orphan nuclear receptor, has been identified to modulate the retinal progenitor cell proliferation and cell cycle re-entry by regulating the expression of PTEN and cyclin D1 (13). Since TLX interacts with atrophin-1, a transcriptional corepressor involved in DRPLA (9, 10). it may be implicated in ophthalmic manifestations of DRPLA (13).

In conclusion, in patients with DRPLA, the presence of corneal endothelial degeneration and/or optic atrophy should be examined using specular micrography and OCT. Depending on the situation, corneal transplantation may be considered one of the treatments for avoiding blindness.

## Data availability statement

The datasets presented in this article are not readily available because of ethical and privacy restrictions. Requests to access the datasets should be directed to the corresponding author.

## Ethics statement

The studies involving human participants were reviewed and approved by the Ethics Committee in Tokai University Hospital. The patients/participants provided their written informed consent to participate in this study. Written informed consent was obtained from the individual(s) for the publication of any potentially identifiable images or data included in this article.

## Author contributions

ST, EN, and WT contributed to the conception and design of the study. ST, KK, YuO, YoO, and HM participated in the clinical management of patients and data collection. ST wrote the first draft of the manuscript. YuO, EN, WT, and HM wrote sections of the manuscript. All authors, especially HM, contributed to manuscript revision, read, and approved the submitted version.

## Conflict of interest

The authors declare that the research was conducted in the absence of any commercial or financial relationships that could be construed as a potential conflict of interest.

## Publisher's note

All claims expressed in this article are solely those of the authors and do not necessarily represent those of their affiliated organizations, or those of the publisher, the editors and the reviewers. Any product that may be evaluated in this article, or claim that may be made by its manufacturer, is not guaranteed or endorsed by the publisher.

## Abbreviations

CV: Coefficient of Variation
DRPLA: Dentatorubral-Pallidoluysian Atrophy
ECD: Endothelial Cell Density
HEX: Hexagonality
MRI: Magnetic Resonance Imaging
OCT: Optical Coherence Tomography
RNFL: Retinal Nerve Fiber Layer.
