# Supplementary material for: Case report: Corneal endothelial degeneration and optic atrophy in dentatorubral-pallidoluysian atrophy quantified by specular micrography and optical coherence tomography
Source: Front Neurol. 2022 Sep 13;13:953787. doi: 10.3389/fneur.2022.953787 (PMC9513026; doi:10.3389/fneur.2022.953787)
Supplement: Supplementary file 1 [file Data_Sheet_1.PDF]

1.1 Supplementary Figures 1

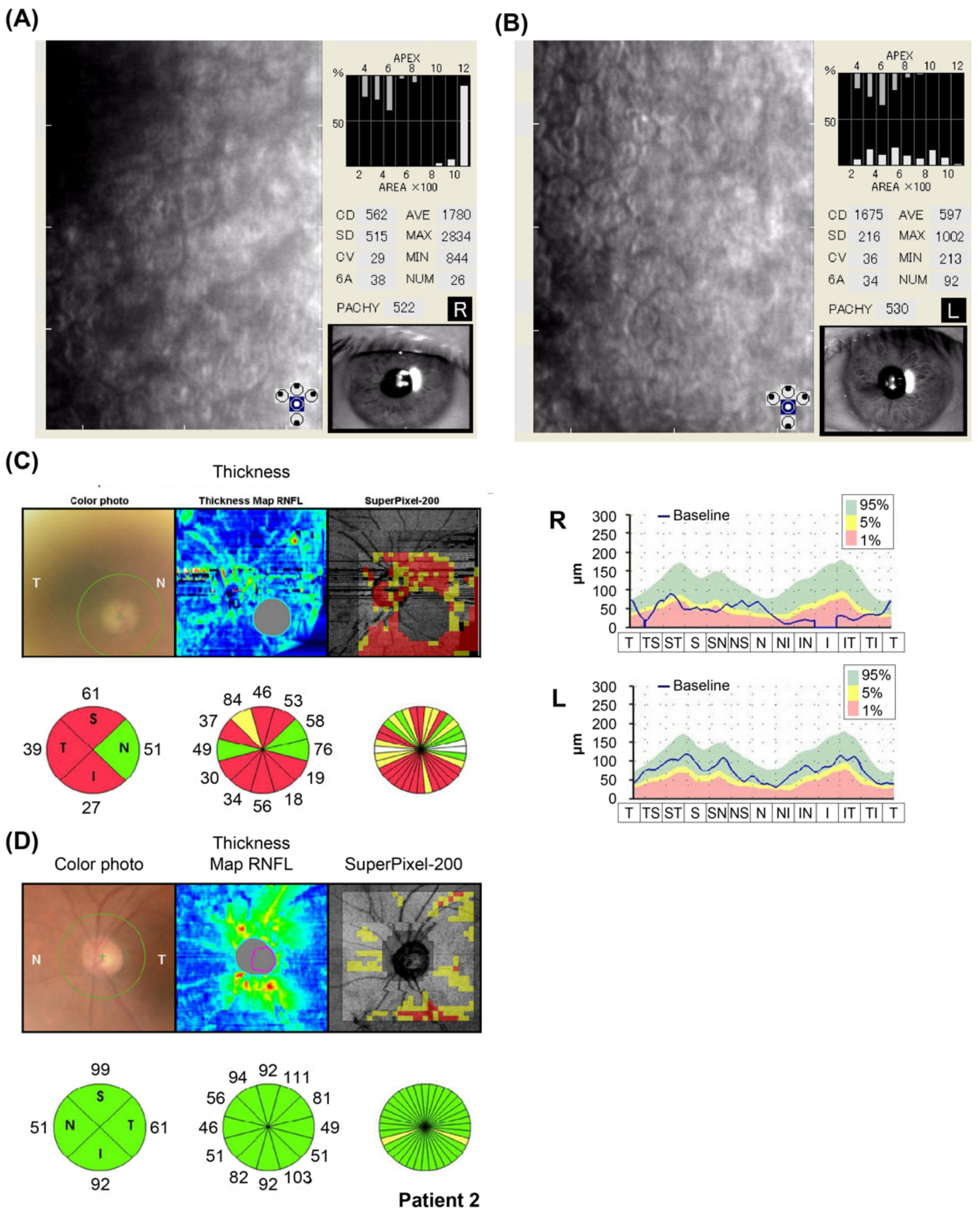

Supplementary Figure 1. Endothelial corneal cell density (ECD) and circumpapillary retinal nerve fiber layer (RNFL) in Patient 2 (Ⅲ-5). Specular microscopy (FA-3609, Konan Medical, Inc. Japan) shows corneal guttata and pleomorphic cellular patterns of endothelial structures in both eyes (A, B). Corneal ECD reduced to 562 and 1675 cells/mm^2 in the right and left eyes, respectively. Optical coherence tomography (3D CT-2000, Topcon Medical Japan, Co. Ltd, Japan) shows RNFL thinning corresponding to optic nerve atrophy in the right eye (C: average RNFL thickness, 46 µm) but not in the left eye (D: average RNFL thickness 76 µm).
