# Supplementary material for: Case report: Corneal endothelial degeneration and optic atrophy in dentatorubral-pallidoluysian atrophy quantified by specular micrography and optical coherence tomography
Source: Front Neurol. 2022 Sep 13;13:953787. doi: 10.3389/fneur.2022.953787 (PMC9513026; doi:10.3389/fneur.2022.953787)
Supplement: Supplementary file 2 [file Data_Sheet_2.PDF]

1.1 Supplementary Figures 2

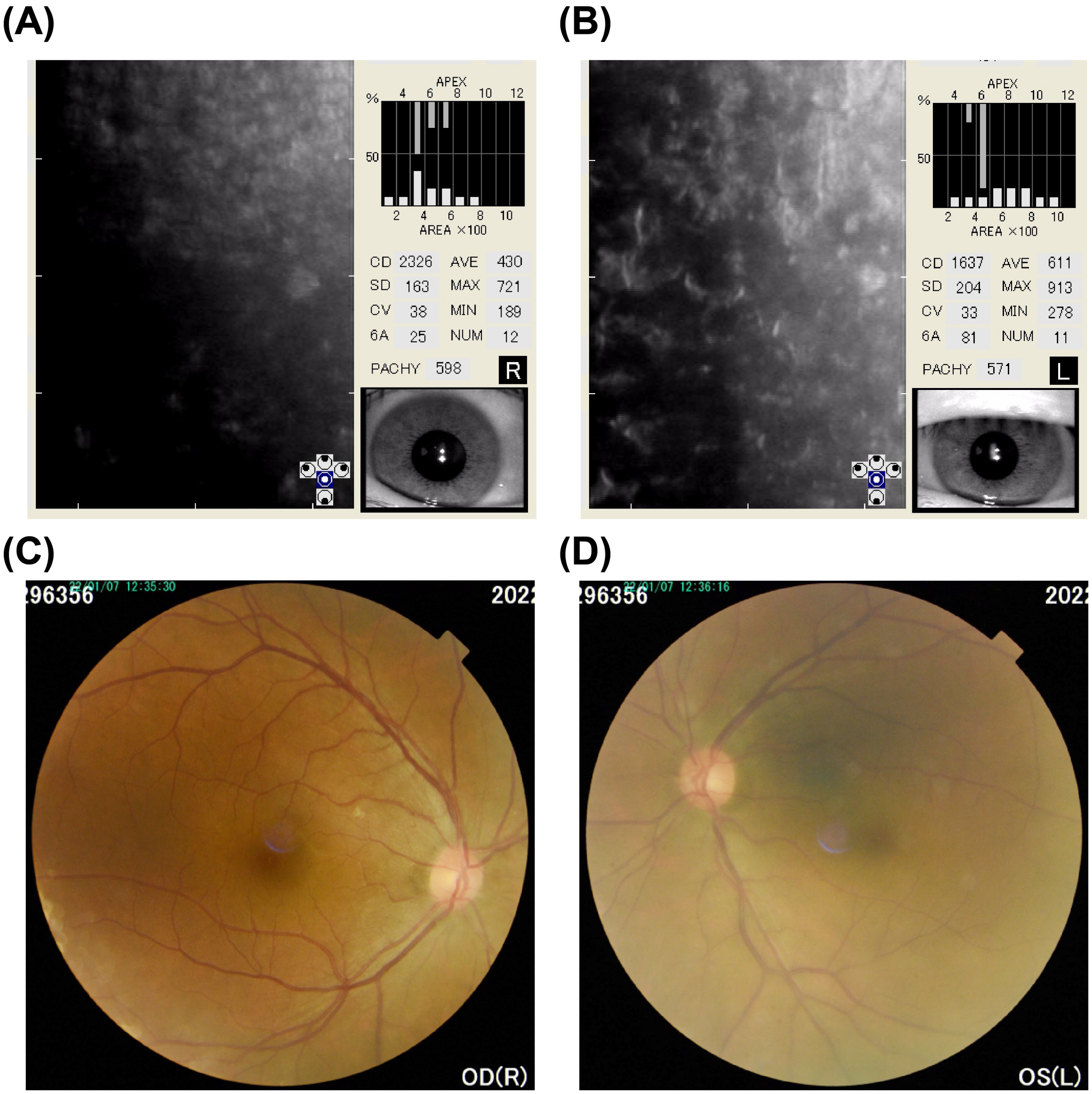

Patient 3

Supplemental figure 2 Endothelial corneal cell density (ECD) and ocular fundi in Patient 3 (Ⅳ-3). Specular microscopy (FA-3609, Konan Medical, Inc. Japan) shows corneal guttata, the pleomorphic cellular pattern of endothelial structures (A, B), and rounded dark cells with light borders (B). Corneal ECD in specular microscopy reduced to 2326 cells/mm^2 and 1637 cells/mm^2 in the right eye and left eyes, respectively (A, B). The ocular fundi show no optic atrophy (C, D).
