# Supplementary material for: Case report: Corneal endothelial degeneration and optic atrophy in dentatorubral-pallidoluysian atrophy quantified by specular micrography and optical coherence tomography
Source: Front Neurol. 2022 Sep 13;13:953787. doi: 10.3389/fneur.2022.953787 (PMC9513026; doi:10.3389/fneur.2022.953787)
Supplement: Supplementary file 3 [file Data_Sheet_3.PDF]

1.1 Supplementary Figures 3

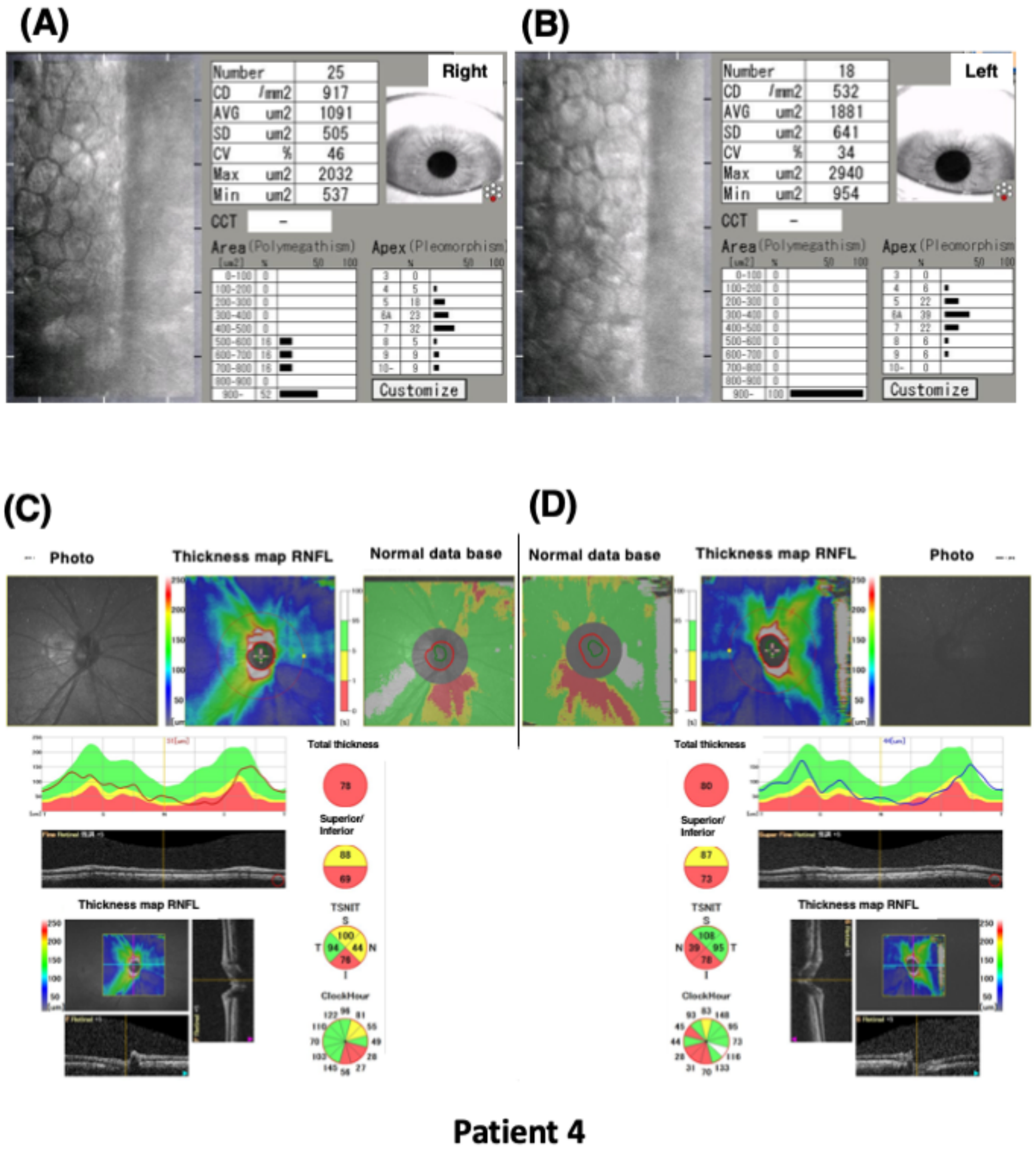

Supplemental figure 3 Endothelial corneal cell density (ECD) and circumpapillary retinal nerve fiber layer (RNFL) in Patient 4 (Ⅳ-1). Specular microscopy (EM-3000, TOMEY, Co. Ltd, Japan) shows the pleomorphic cellular pattern of endothelial structures (A) and rounded dark cells with light borders (A). Corneal ECD in specular microscopy reduced to 917 cells/mm^2 and 532 cells/mm^2 in the right eye (A) and left eye (B), respectively. Although OCT (RS-3000 advance, NIDEK Co., Ltd, Japan) shows thinning of RNFL in the inferior area of the optic nerve papilla in the right (C) and left eyes (D), this finding is not reliable because the optic disc is tilted due to high myopia.
